# A dualistic model of primary anal canal adenocarcinoma with distinct cellular origins, etiologies, inflammatory microenvironments and mutational signatures: implications for personalised medicine

**DOI:** 10.1038/s41416-018-0049-2

**Published:** 2018-04-27

**Authors:** Michael Herfs, Patrick Roncarati, Benjamin Koopmansch, Olivier Peulen, Diane Bruyere, Alizee Lebeau, Elodie Hendrick, Pascale Hubert, Aurelie Poncin, William Penny, Nathalie Piazzon, Franck Monnien, David Guenat, Christiane Mougin, Jean-Luc Prétet, Lucine Vuitton, Karin Segers, Frederic Lambert, Vincent Bours, Laurence de Leval, Severine Valmary-Degano, Charles M Quick, Christopher P Crum, Philippe Delvenne

**Affiliations:** 1 Laboratory of Experimental Pathology, GIGA-Cancer, University of Liege, 4000 Liege, Belgium; 2 Department of Human Genetics, University Hospital Center of Liege, 4000 Liege, Belgium; 3 Metastasis Research Laboratory, GIGA-Cancer, University of Liege, 4000 Liege, Belgium; 4 Department of Medical Oncology, University Hospital Center of Liege, 4000 Liege, Belgium; 5 Laboratory of Human Genetics, GIGA-Cancer, University of Liege, 4000 Liege, Belgium; 6 Department of Pathology, University of Arkansas for Medical Sciences, Little Rock, AR 72205 USA; 7 Institute of Pathology, Lausanne University Hospital, University of Lausanne, 1011 Lausanne, Switzerland; 8 Department of Pathology, Besançon University Hospital, University of Bourgogne Franche-Comté, 25000 Besançon, France; 9 EA3181, University of Bourgogne Franche-Comté, LabEx LipSTIC ANR-11-LABX-0021, 25000 Besançon, France; 10 CNR Papillomavirus, Besançon University Hospital, 25000 Besançon, France; 11 Department of Medicine, Division of Oncology, Stanford Cancer Institute, Stanford University, Stanford, CA 94304 USA; 12 Department of Gastroenterology, Besançon University Hospital, University of Bourgogne Franche-Comté, 25000 Besançon, France; 13 Department of Pathology, Division of Women’s and Perinatal Pathology, Brigham and Women’s Hospital, Harvard Medical School, Boston, MA 02215 USA

**Keywords:** Anal cancer, Tumour virus infections

## Abstract

**Background:**

Primary adenocarcinoma of the anal canal is a rare and aggressive gastrointestinal disease with unclear pathogenesis. Because of its rarity, no clear clinical practice guideline has been defined and a targeted therapeutic armamentarium has yet to be developed. The present article aimed at addressing this information gap by in-depth characterising the anal glandular neoplasms at the histologic, immunologic, genomic and epidemiologic levels.

**Methods:**

In this multi-institutional study, we first examined the histological features displayed by each collected tumour (*n* = 74) and analysed their etiological relationship with human papillomavirus (HPV) infection. The intratumoural immune cell subsets (CD4, CD8, Foxp3), the expression of immune checkpoints (PD-1, PD-L1), the defect in mismatch repair proteins and the mutation analysis of multiple clinically relevant genes in the gastrointestinal cancer setting were also determined. Finally, the prognostic significance of each clinicopathological variable was assessed.

**Results:**

Phenotypic analysis revealed two region-specific subtypes of anal canal adenocarcinoma. The significant differences in the HPV status, density of tumour-infiltrating lymphocytes, expression of immune checkpoints and mutational profile of several targetable genes further supported the separation of these latter neoplasms into two distinct entities. Importantly, anal gland/transitional-type cancers, which poorly respond to standard treatments, displayed less mutations in downstream effectors of the EGFR signalling pathway (i.e., *KRAS* and *NRAS)* and demonstrated a significantly higher expression of the immune inhibitory ligand-receptor pair PD-1/PD-L1 compared to their counterparts arising from the colorectal mucosa.

**Conclusions:**

Taken together, the findings reported in the present article reveal, for the first time, that glandular neoplasms of the anal canal arise by HPV-dependent or independent pathways. These etiological differences leads to both individual immune profiles and mutational landscapes that can be targeted for therapeutic benefits.

## Introduction

Extending from the anal margin (also called perianal skin) to the anorectal ring/flexure, the anal canal is the terminal part of the gastrointestinal tract. Despite its small size (~4 cm in length), several types of neoplams may be observed within this anatomical structure, which reflects its embryologic/histologic complexity.^1^ Accounting for ~90% of all malignant anal lesions, squamous cell carcinomas (SCC) arise either from the external (squamous) part of the anal canal or from the transitional zone lined by an “urothelium-like” epithelium.^2,3^ Importantly, the cellular origin was recently shown to strongly impact the protein expression profile, the differentiation as well as the outcome of these tumours.^2^ Etiologically linked to HPV infection (most notably HPV16 genotype),^2,4–6^ SCC are traditionally treated by surgery (local resection) and/or chemoradiotherapy (radiation therapy combined with mitomycin C and 5-Fluorouracil). With the exception of rare HPV-negative anal SCC (~5%) that are unresponsive to these standard treatments,^2,5,6^ a complete remission is achieved in the majority (~75%) of patients.^7^ Beside squamous neoplasms, a few thousands of primary anal canal adenocarcinoma are also diagnosed each year worldwide. More aggressive than SCC and most frequently detected in older patients (sixth decade of life),^8–11^ these rare tumours still represent a diagnostic and therapeutic challenge due to the lack of in-depth characterisation. According to a survey conducted by Abel et al.,^12^ most clinicians only occasionally encounter patients with anal adenocarcinoma. Therefore, the majority of recent descriptive studies are clinical case reports,^13–15^ and there is a lack of sufficient information to generate guidelines for uniform treatment recommendations.^16,17^ Moreover, molecular perturbations that might serve as targets for therapy are completely unknown.

In the present study, we extensively examined a multicentre cohort of anal canal adenocarcinoma (*n* = 74) in order to improve the current controversial diagnosis/management of patients. The histologic, immunologic and mutational profiles of these rare malignant glandular lesions were, for the first time, determined and collected results point to new opportunities for personalised therapeutic intervention.

## Materials and methods

### Patient selection and clinical data retrieval

A total of 74 patients treated for primary anal canal adenocarcinoma in six different medical centres (University Hospital Center of Liege (Belgium), Citadelle Regional Hospital (Liege, Belgium), Jules Bordet Institute (Brussels, Belgium), University Hospital Center of Besançon (France), University Hospital of Lausanne (Switzerland) and University of Arkansas for Medical Sciences (Little Rock, AR, USA)) between January 1999 and October 2016 were selected. Tissue specimens (biopsies or tumour resections) from each patient were retrieved from pathology archives with the approval of the ethics committees of the respective institutions. Forty normal tissue samples (haemorrhoidectomies) displaying the three different portions of the anal canal (squamous zone, transitional zone and colorectal zone) were also obtained. All cases (haematoxylin and eosin (H&E) staining) were re-examined by senior histopathologists to confirm the diagnosis. Clinicopathological data were collected for all selected patients diagnosed with invasive adenocarcinoma of the anal canal. Patient gender, age at diagnosis, disease stage (according to the Union for International Cancer Control, 8th edition, 2016), HIV status, inflammatory bowel disease, treatment details [local surgery, (neo)adjuvant therapies (radiotherapy and/or chemotherapy), abdomino-perineal resection] and follow-up data were obtained from patient’s medical records. Tumour differentiation was determined using established criteria (Supplementary Figure 1). Patients with downward spread from a rectal tumour, associated with incomplete clinicopathological information or treated for a glandular neoplasm from questionable origin were excluded.

### Immunohistochemistry and immunostaining assessment

Immunohistochemical analyses were performed using a standard protocol extensively described previously.^2,18–20^ The following antibodies were used for the primary reaction: anti-keratin (Krt) 7 (clone SP52; Ventana Medical Systems, Tucson, AZ, USA), anti-Krt16 (clone LL025, Thermo Scientific, Rockford, IL, USA), anti-Krt20 (clone SP33; Ventana Medical Systems), anti-CDX2 (clone EPR2764Y; Ventana Medical Systems), anti-p16^ink4^ (clone JC8; Santa Cruz Biotechnology, Santa Cruz, CA, USA), anti-Ki67 (clone Mib-1; Dako, Glostrup, Denmark), anti-CD4 (clone SP35; Ventana Medical Systems), anti-CD8 (clone SP57; Ventana Medical Systems), anti-PD-1 (clone NAT105; Abcam, Cambridge, MA, USA), anti-PD-L1 (clone 28-8; Abcam), anti-Foxp3 (clone 236 A/E7; eBioscience, San Diego, CA, USA) and anti-EGFR (clone 5B7, Ventana Medical Systems). The mouse/rabbit Envision (Dako) or Novolink polymer (Leica Biosystems, Wetzlar, Germany) detection systems were used for the secondary reaction. Mouse and rabbit control IgGs (Santa Cruz Biotechnology) were utilised as negative controls.

All immunolabelled tissues were evaluated independently by experienced histopathologists. Krt7, Krt16, Krt20 and CDX2 immunostainings were scored as positive when a strong/uniform immunoreactivity was detected in more than 90% of epithelial cells. The growth fraction (proliferative index) of a given tumour was determined with the percentage of Ki67-positive cells. Collected results were stratified as follows: 0–5%, 6–25%, 26–50%, 51–75%, and* > *75%. A similar scoring stratification was used to assess nuclear/cytoplasmic p16^ink4^ immunoreactivity. T cell subsets (CD4^+^, CD8^+^, Foxp3^+^ and PD-1^+^) infiltrating the epithelial component of the tumour or the stroma surrounding cancer cells were quantified by computerised counts (QuPath 0.1.2 open source software for digital pathology image analysis) and verified by manual counting. The number of positive cells was reported to tumour area yielding a count expressed as number of cells/mm^2^. As previously described,^19,21^ both PD-L1 and EGFR immunolabelled tissues were evaluated by using a semi-quantitative score of the intensity (0: undetectable, 1: low, 2: moderate, 3: strong) and extent (0:<5% positive cells, 1: 6–33%, 2: 34–66%, 3: >67%) of the staining, according to an arbitrary scale. The results obtained with these two scales were multiplied in order to obtain a global score ranged between 0 and 9 for each specimen.

### HPV genotyping and physical status

The simultaneous detection of 14 carcinogenic (high-risk) HPV genotypes (16, 18, 31, 33, 35, 39, 45, 51, 52, 56, 58, 59, 66, and 68) was performed using the Abbott RealTime High-Risk HPV assay (Abbott, Wiesbaden, Germany). As previously demonstrated,^22^ this test is highly sensitive for detecting HPV infection in paraffin-embedded tissues.

The HPV16 and 18 physical status (episomal, mixed, integrated) was determined by analysing the disruption of the HPV E2 gene expression by quantitative real-time PCR (ABI-Prism 7900 HT Sequence Detection System, Applied Biosystems, Foster City, CA, USA). Both thermal cycling conditions and primer sequences are available in Supplementary materials and methods. Each experiment was performed in triplicate and normalised to the amount of GAPDH mRNA from the same sample. The E6/E2 ratio cut-off value was determined, as previously described.^23^

### In situ hybridisation

DNA in situ hybridisation was performed according to the manufacturer’s instructions. The most common carcinogenic HPV genotypes (16, 18, 31, 33, 35, 39, 45, 51, 52, 56, 58, and 66) were detected using a probe cocktail (INFORM HPV III Family 16 Probe, Ventana Medical Systems). Red Counterstain II (Ventana Medical System) was used in order to facilitate microscopic observation.

### Microsatellite instability (MSI) assessment

The MSI status of tumour specimens was determined using the pentaplex PCR assay described by Buhard et al. and comprising 5 quasimonomorphic mononucleotide repeats (NR-27, NR-21, NR-24, BAT-25, and BAT-26).^24,25^ Each collected tissue sample was carefully reviewed by a histopathologist in order to ascertain that the percentage of neoplastic cell was higher than 40%. When cancer cell enrichment was necessary, tumour areas were macrodissected with a scalpel before tissue digestion. Genomic DNA extraction, PCR conditions and primer sequences are detailed in Supplementary materials and methods. PCR products labeled with fluorescent dyes were analysed with an ABI 3500xL Genetic Analyzer (Applied Biosystems). As recommended by the revised Bethesda guidelines,^26^ tumours displaying differences in the length of two or more microsatellite sequences (markers) were interpreted as being MSI-High. Lesions showing no instability or one instable repeat were defined as microsatellite stable (MSS) and MSI-Low, respectively.

### Mutation analysis using next-generation sequencing (NGS)

As described above, all analysed samples contained at least 40% tumour cells. Both tissue lysis and DNA extraction were performed using QIAamp DNA FFPE Tissue Kit (Qiagen, Valencia, CA, USA) according to supplier’s recommendations. Frequently mutated regions of 10 clinically relevant genes in the gastrointestinal cancer setting [*KRAS* [exons 2, 3 and 4 (full)], *NRAS* [exons 2, 3 and 4 (full)], *BRAF* [exons 11 and 15 (full)], *PIK3CA* [hotspots in exon 9 and 20 (codons 542, 545 and 1047 covered)], *EGFR* [exons 18, 19, 20 and 21 (full)], *HER2* [exon 22 (full)], *KIT* [exons 9, 11, 13, 14, 17 and 18 (full)], *PTEN* [exons 5 and 7 (full)], *PDGFRA* [exons 12, 14 and 18 (full)], *DDR2* [exon 18 (full)]] were amplified and sequenced using NGS technology. The detailed protocol is available in Supplementary materials and methods.

### Methylation-specific PCR (MSP)

DNA isolated from anal tissue specimens were subjected to bisulfite treatment according to the manufacturer’s instructions (Promega, Madison, WI, USA). Both detailed procedure and primer sequences targeting *p16*^*ink4*^ promoter were previously described.^27^ PCR products were loaded on 2% agarose gels, stained with ethidium bromide and visualised with an UV transilluminator (Bio-Rad, Hercules, CA, USA).

### Statistical analysis

All collected data were processed using the R (version 3.3.3) or GraphPad Prism 5 software packages. Differences were considered statistically significant when *p < *0.05. A two-tailed Student’s *t*-test was used to determine the statistical difference between two groups of immunostained specimens. The comparison of clinicopathological data between independent groups was performed by using Fisher’s exact test or a χ^2^ test. Disease-free (DFS) and overall (OS) survivals were defined as the time between the original diagnosis/biopsy and the date of recurrence (at the primary or distant site) or death (from any cause), respectively. When the event did not occur, the date of the last follow-up visit was used as the endpoint. The Kaplan–Meier methods was used to estimate both the OS and DFS. For multivariate analysis of clinicopathological variables related to outcome, a COX regression model was performed in which significant risk factors in univariate analysis were included (in this particular case, a cutoff at *p* < 0.10 was used).

## Results

### Region-specific proteins reveal two distinct subtypes of primary adenocarcinoma of the anal canal

**Fig. 1:**
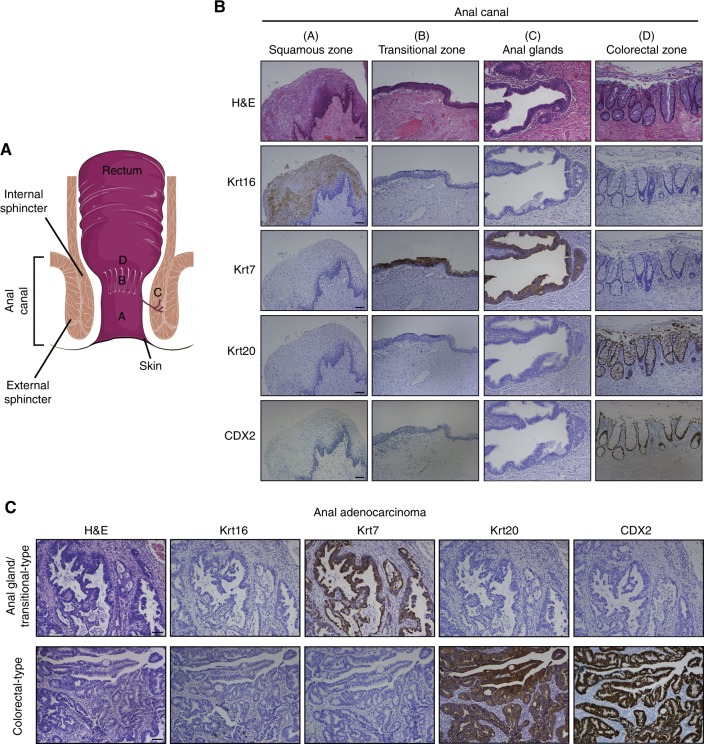
Identification of two distinct subtypes of anal adenocarcinoma. **a** Schematic representation of the anal canal. **b** Histology of the different parts of the anal canal and characterisation of several region-specific biomarkers (Krt16: squamous zone; Krt7: anal glands/transitional zone; Krt20 and CDX2: colorectal zone). **c** Phenotypic analyses reveal two region-specific subpopulations of primary anal canal adenocarcinoma. Note the diffuse Krt7 immunoreactivity displayed by tumours arising from the anal glands/transitional zone. In contrast, colorectal-type anal adenocarcinoma strongly expressed both Krt20 and CDX2 and stained negative for Krt7. The scale bar represents 100 μm

The anal canal can be histologically divided into three parts: (1) the external zone lined by a squamous epithelium, (2) the transitional zone covered by an “urothelium-like” epithelium and (3) the colorectal zone. Anal glands, responsible for mucus secretion, open directly in the transitional zone (Fig. 1a). The microscopic detection of hair follicles and/or sweat glands delineates the perianal skin (anal margin) from the squamous portion of the anal canal. In contrast, similar to the rectum, the upper centimeter of the anal canal is lined by a colorectal epithelium preventing a clear demarcation of these two structures by microscopy. Therefore, medical imaging modalities (endoscopic ultrasound, computed tomography scan and/or magnetic resonance imaging) were used to ascertain the anal origin of all selected specimens. We previously performed proteomic analysis of micro-dissected paraffin-embedded tissues and discovered biomarkers that demarcated the different portions of the normal anal canal.^2^ As shown in Fig. 1b, Krt16 is specifically expressed in the squamous zone, whereas Krt7 is only detected in the anal transitional zone/anal glands. As for Krt20 and CDX2, their simultaneous expressions highlight the colorectal epithelium. In the present study, the robustness of these biomarkers was first assessed with 40 normal anal canal specimens and a 100% specificity/sensitivity was observed for all of them. All collected primary adenocarcinoma of the anal canal were then immunostained and the expression profile of region-specific proteins clearly showed two distinct subtypes of glandular neoplasms (Fig. 1c). Out of 74 cancer specimens, 26 (35.1%) displayed a diffuse Krt7 immunoreactivity and an absence of Krt20/CDX2 expression. The opposite pattern was observed in “colorectal-type” anal adenocarcinoma which were more frequently detected (48/74, 64.9%) compared to their counterparts arising from the anal glands/transitional zone. In agreement with the columnar differentiation displayed by all these tumours, the expression of Krt16 (Fig. 1c) and other squamous markers such as p63 and Krt14 (data not shown) was never detected. As illustrated in Supplementary Figure 2, a minority of tumours originated from the colorectal zone of the anal canal were negative for Krt20 (4/48, 8.3%) or displayed a patchy Krt7 immunoreactivity (5/48, 10.4%).

### Almost half of glandular neoplasms arising from the anal glands/transitional zone are etiologically linked to high-risk HPV infection

**Fig. 2:**
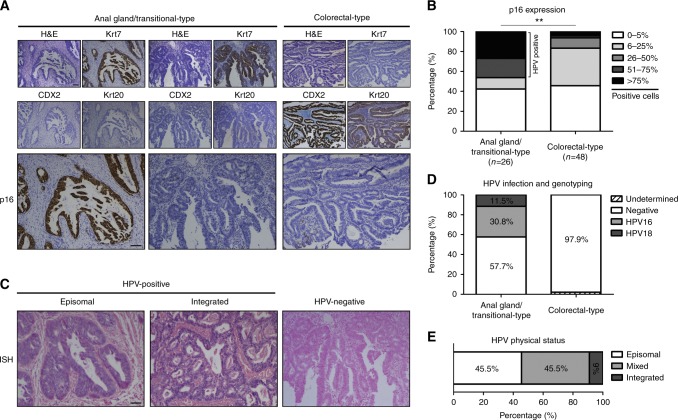
HPV16/18 DNA and viral oncogene expression are detected in a significant proportion of anal gland/transitional zone-type adenocarcinoma. **a** Representative examples of primary adenocarcinoma of the anal canal stained for p16^ink4^. **b** Semi-quantitative evaluation of this surrogate biomarker for HPV infection in both anal gland/transitional zone-type and colorectal-type cancers. Note the strong/diffuse p16^ink4^ immunoreactivity observed in approximately half (12/26, 46.2%) of Krt7-positive tumours. **c** Anal glandular neoplasms displaying staining patterns of episomal or integrated HPV DNA (in situ hybridisation). **d** HPV genotypes identified in our cohort. **e** Physical status of viral infection in HPV-positive anal adenocarcinoma specimens (identified by E2/E6 ratio analysis). The scale bar represents 100 μm. Asterisks indicate statistically significant differences (**p < 0.01)

Carcinogenic HPV genotypes are detected in most (~95%) anal squamous (pre)neoplastic lesions. To examine the possible etiological link between HPV infection and glandular carcinoma development within the anal canal, we first analysed the expression of p16^ink4^ (a surrogate biomarker for high-risk HPV detection) in our cohort of anal adenocarcinoma. Representative examples are shown in Fig. 2a. A strong and diffuse p16^ink4^ immunoreactivity (>50% positive cells) was observed in 15 invasive cancers [12 (12/26, 46.2%) anal gland/transitional zone-type adenocarcinoma and 3 (3/48, 6.3%) malignant lesions arising from the colorectal zone] (Fig. 2b). The presence of HPV was confirmed by both in situ hybridisation (Fig. 2c) and PCR (Fig. 2d) in 11 (11/26, 42.3%) Krt7-positive malignancies. HPV16 and 18 genotypes were identified in 8 (72.7%) and 3 (27.3%) cases, respectively. No multiple infection was observed. In contrast, HPV DNA was not detected in any of the colorectal-type anal adenocarcinoma. Because of insufficient extracted DNA, the HPV status was undetermined in 1 patient (1/48, 2.1%) with colorectal-type anal adenocarcinoma. In order to determine the physical status of HPV infection, E2 and E6 gene expression was evaluated by real-time PCR. In view of collected E2/E6 ratios, HPV DNA was exclusively episomal in 5 cases (5/11, 45.5%), mixed in 5 tissue samples (5/11, 45.5%) and integrated in 1 specimen (1/11, 9%) (Fig. 2e). These latter results correlated with the staining patterns found by in situ hybridisation. Altogether, these findings were construed as direct evidence for a transcriptionally active HPV infection in around half of primary anal adenocarcinomas originating from the anal glands/transitional zone.

### Several clinicopathological characteristics of patients vary according to the micro-anatomical origin of tumours

Clinicopathological data from 74 patients with histologically confirmed anal adenocarcinoma and treated between January 1999 and October 2016 were collected from 6 University Hospital Centers. As mentioned above, based on medical imaging results, all these glandular tumours were strictly located within the anal canal. Overall, patients were relatively aged (mean: 67, ranged from 36 to 94 years) and males slightly outnumbered females (male/female ratio: 1.24). Approximately half (38/74, 51.4%) of patients had a lymphatic node involvement and 9 (12.2%) patients had distant metastasis (mainly in the liver (6/9, 66.7%)) at presentation. Most patients (42/74, 56.8%) underwent surgery (abdominoperitoneal resection or local excision) with neoadjuvant or postoperative chemoradiotherapy. The prescribed radiotherapy dose was usually 45 Gy (in 25 fractions) and 5-Fluorouracil was the most commonly used chemotherapeutic drug. A few patients also received FOLFOX (Folinic acid, 5-Fluorouracil and Oxaliplatin) (9/74, 12.2%) (mainly as adjuvant treatment) or Capecitabine (Xeloda) (4/74, 5.4%). As listed in Table 1, when tumour origin (determined by the Krt7/20-CDX2 expression profile) was taken into account for subclassifying patients into two groups, no significant difference was noted with respect of age at diagnosis (*p* = 1), gender (*p* = 1), tumour differentiation (*p = *0.313), TNM classification (T, *p* = 0.339; N, *p* = 1; M, *p* = 0.479), tumour stage (*p* = 0.847) and treatment (*p* = 0.062). No patient was HIV positive. As mentioned earlier, high-risk HPV infection (HPV16 or HPV18) was only detected in neoplastic lesions arising from the anal glands/transitional zone (*p < *0.001). Significantly, these latter tumours displayed a lower proliferative index compared to their counterparts originating from the colorectal mucosa (*p* = 0.003). Crohn’s disease and ulcerative colitis were also statistically correlated with tumour origin (*p = *0.049). With the exception of one case (1/48, 2.1%), all these inflammatory conditions were observed in patients with anal gland/transitional zone-type tumours (4/26, 15.4%). Three out of 4 (75%) were associated with chronic perianal fistulas. Finally, patients’ characteristics for Krt7-positive anal adenocarcinoma according to HPV status are shown in Supplementary Table 1. Although HPV-positive tumours tended to be diagnosed in younger patients compared to uninfected cancers, similarly to all other clinicopathological variables, statistical significance was not reached. These results might be related to the weak number of patients in each category (lack of statistical power).

**Table 1:** Demographic and patient characteristics according to tumour subtype/origin

Characteristics	Anal gland/transitional-type (*n* = 26) (35.1%)	Colorectal-type (*n* = 48) (64.9%)	*P*-value
Age at diagnosis (mean: 67) (range: 36–94) (years)			1
<65	12 (46.2%)	22 (45.8%)	
≥65	14 (53.8%)	26 (54.2%)	
Gender			1
Male	14 (53.8%)	27 (56.3%)	
Female	12 (46.2%)	21 (43.7%)	
Inflammatory bowel disease			**0.049**
Negative	22 (84.6%)	47 (97.9%)	
Positive	4 (15.4%)	1 (2.1%)	
HIV infection			/
Negative	26 (100%)	48 (100%)	
Positive	0 (0%)	0 (0%)	
HPV infection			**<0.001**
Negative	15 (57.7%)	47 (97.9%)	
Positive	11 (42.3%)	0 (0%)	
Undetermined	0 (0%)	1 (2.1%)	
HPV genotypes			/
HPV16	8 (72.7%)	0 (0%)	
HPV18	3 (27.3%)	0 (0%)	
Others	0 (0%)	0 (0%)	
Proliferative index (Ki67)			**0.003**
≤25%	7 (26.9%)	4 (8.3%)	
26–50%	11 (42.3%)	10 (20.9%)	
51–75%	7 (26.9%)	17 (35.4%)	
>75%	1 (3.9%)	17 (35.4%)	
Tumour differentiation			0.313
Well-differentiated	9 (34.6%)	25 (52.2%)	
Moderately differentiated	13 (50%)	16 (33.3%)	
Poorly differentiated	4 (15.4%)	7 (14.5%)	
cTNM			
cT			0.339
T1–T2	14 (53.8%)	20 (41.7%)	
T3–T4	12 (46.2%)	28 (58.3%)	
cN			1
N−	13 (50%)	23 (47.9%)	
N+	13 (50%)	25 (52.1%)	
cM			0.479
M−	24 (92.3%)	41 (85.4%)	
M+	2 (7.7%)	7 (14.6%)	
Tumour stage (UICC)			0.847
Stage I	2 (7.7%)	3 (6.3%)	
Stage II	11 (42.3%)	20 (41.7%)	
Stage III	11 (42.3%)	18 (37.5%)	
Stage IV	2 (7.7%)	7 (14.5%)	
Primary treatment			0.062
(neo)adjuvant chemoradiotherapy/surgery	11 (42.3%)	31 (64.6%)	
(neo)adjuvant radiotherapy/surgery	4 (15.4%)	1 (2.1%)	
(neo)adjuvant chemotherapy/surgery	1 (3.9%)	1 (2.1%)	
Chemoradiotherapy	2 (7.7%)	0 (%)	
Radiotherapy	3 (11.5%)	3 (6.3%)	
Chemotherapy	2 (7.7%)	1 (2.1%)	
Surgery	3 (11.5%)	7 (14.5%)	
No treatment	0 (0%)	4 (8.3%)	

Bold values indicate statistically significant differences

### Patients treated for anal gland/transitional-type or colorectal-type anal adenocarcinoma undergo frequent recurrences and are associated with a similar likelihood of adverse outcome

**Fig. 3:**
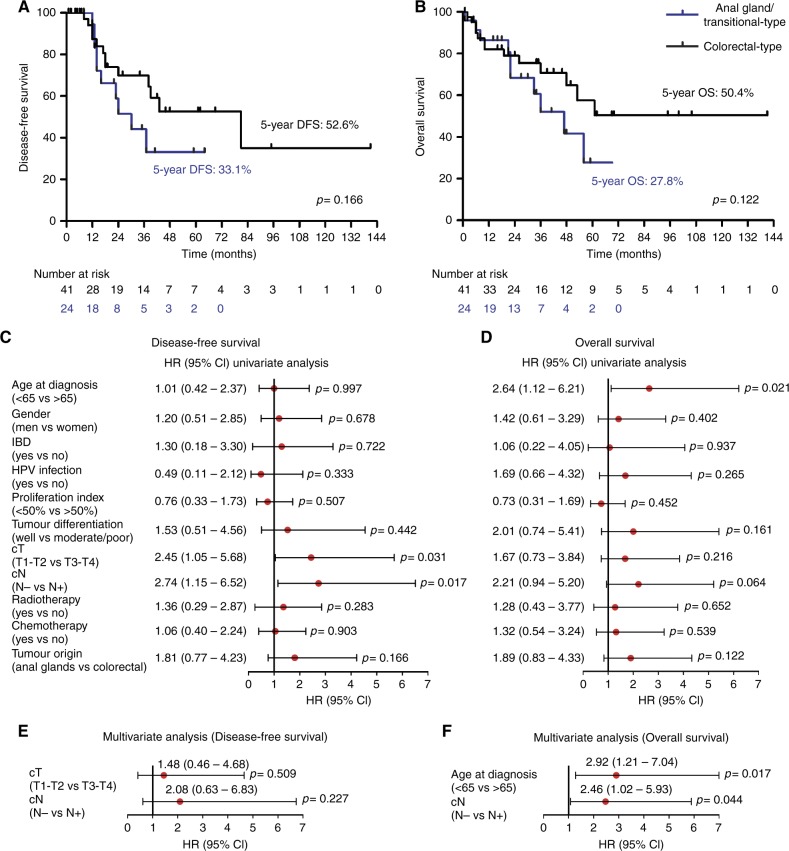
Outcome of patients according to tumour origin (determined by Krt7 and Krt20/CDX2 expression pattern). **a** Disease-free survival (DFS) and **b** overall survival for both region-specific subgroups of anal adenocarcinoma. Prognostic value of clinicopathological parameters (risk factors) in univariate (**c**, **d**) and multivariate (**e**, **f**) analysis for DFS and OS

Out of 74 patients diagnosed with adenocarcinoma strictly located in the anal canal, 65 (87.8%) were incorporated in both OS and DFS analysis. Nine (12.2%) patients were excluded because of distant metastasis at the time of diagnosis (UICC stage IV). Furthermore, 5 (55.6%) of these latter metastatic patients did not receive any treatment. Median follow-up was 33.1 months (range: 2–141 months). After treatment completion, local or distant recurrence occurred in 10 (10/24, 41.7%) patients with invasive neoplasms originating from the anal glands/transitional zone and in 13 (13/41, 31.7%) patients with colorectal-type anal adenocarcinoma. The two tumour subtypes displayed similar patterns of local/metastatic recurrences and, whatever the tumour origin, all distant recurrences were found either in the liver or the lung. Among the patients who died during the follow-up period, 11 (11/24, 45.8%) and 13 (13/41, 31.7%) exhibited a neoplastic lesion who developed from the anal glands/transitional zone or from the colorectal mucosa anatomically located in the anal canal, respectively. Cancer was the cause of the death in three quarter of cases (18/24, 75%). As illustrated by the Kaplan-Meier survival curves (Fig. 3a, b), patients with primary anal adenocarcinoma frequently experienced a poor outcome. In particular, patients treated for anal gland/transitional-type cancer tended to have shorter 5-years DFS (33.1% versus 52.6%) and OS (27.8% versus 50.4%) compared with those with a colorectal-type neoplasm. Statistical significance was not reached (Fig. 3c–f).

### Age at diagnosis, nodal status and tumour size significantly impact the outcome of patients treated for anal adenocarcinoma

In order to better predict the outcome of patients, the prognostic values of each clinicopathological variable (risk factor) associated with DFS and OS were then determined. Forest plots for both univariate and multivariate analysis are shown in Fig. 3c–f. Both DFS and OS did not significantly differ with patient gender, concomitant inflammatory condition, HPV status, proliferative index, tumour differentiation, chemotherapy use and radiotherapy (Fig. 3c–d). In univariate analysis, N-stage positivity was significantly related to reduced DFS (*p* = 0.017) (a tendency was also observed with OS (*p* = 0.064)) whereas tumour size (*p* = 0.031) and age at diagnosis (*p* = 0.021) were independently associated with reduced DFS and OS, respectively. The prognostic value of patient age (*p* = 0.017) and N-stage (*p* = 0.044) for OS was further confirmed by multivariate analysis (Fig. 3e–f). When all the parameters (irrespective of the significance in univariate analysis) were included in multivariate analysis, no other significant risk factor was revealed.

### Region-specific subtypes of anal adenocarcinoma display different mutational patterns for downstream effectors of the EGFR signalling pathway

**Fig. 4:**
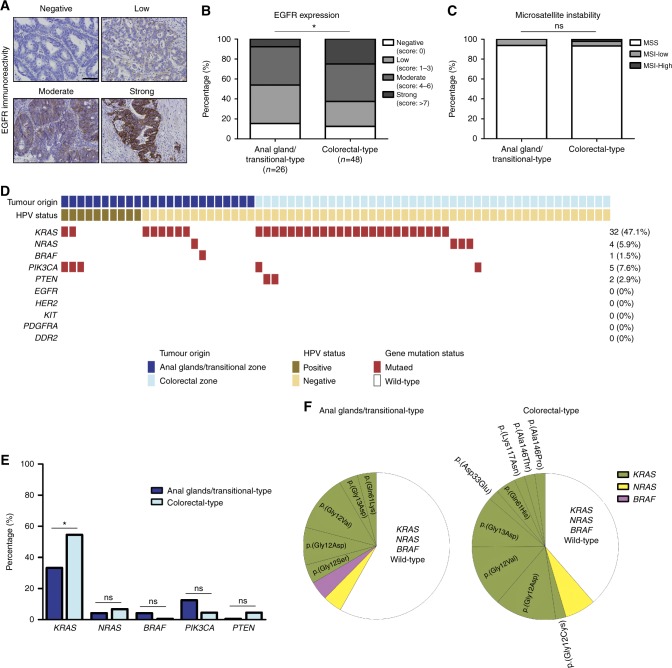
EGFR expression profile in anal adenocarcinoma and mutational status of several clinically relevant genes in the gastrointestinal cancer setting. **a** Representative examples of EGFR immunoreactivity displayed by anal glandular neoplasms. **b** Semiquantitative evaluation of EGFR expression in both anal gland/transitional-type (*n* = 26) and colorectal-type (*n* = 48) anal adenocarcinoma. **c** Microsatellite instability (MSI) status of tumours according to tumour origin. **d** Heat-map representation of individual mutations identified in anal adenocarcinomas. Because of poor DNA quality, 6 specimens out of 74 were not taken into account. **e** Prevalence of neoplasic lesions harboring mutated downstream effectors of the EGFR signalling pathway according to tumour origin. **f** Prevalence of *KRAS*, *NRAS* and *BRAF* mutations and corresponding amino acid sequence alterations. The scale bar represents 100 μm. Asterisks indicate statistically significant differences (*p < 0.05)

As described above, both subtypes of anal adenocarcinoma are globally associated with an unfavorable outcome. In order to offer treatment alternatives that might improve the poor existing 5-year survival rate and reduce the frequency of local/distal recurrences (Fig. 3), it is essential to have a precise characterisation of the disease (expression of targetable proteins, genetic underpinnings, immune microenvironment,…). We first investigated EGFR expression by immunohistochemistry in both subtypes of anal columnar lesions identified in the present study. Representative examples of EGFR immunolabelled tissues are shown in Fig. 4a. Overall, EGFR expression was detected in the large majority of cancers (64/74, 86.5%). With the exception of one case, all stage III and IV tumours were positive (37/38, 97.4%). When both the intensity and the extent of the staining were taken into account, EGFR expression was significantly higher in colorectal-type adenocarcinoma compared to anal gland/transitional-type neoplasms (Fig. 4b). In the last decade, significant improvement in the treatment of several cancers (i.e., metastatic colorectal cancer, non-small cell lung cancer or head and neck SCC) has been obtained with the use of monoclonal anti-EGFR antibodies. However, somatic mutations in one or several downstream effector(s) of the EGFR signalling pathway were clearly shown to be associated to resistance to anti-EGFR drugs (cetuximab and panitumumab).^28,29^ Therefore, the prevalence rates of *KRAS*, *NRAS*, *BRAF*, *PIK3CA*, *EGFR*, *HER2* and *PTEN* mutations were determined in all collected anal adenocarcinoma specimens. The mutational status of three other clinically relevant genes in the gastrointestinal cancer setting (*KIT*, *PDGFRA* and *DDR2*) was also assessed. Because of poor DNA quality (high degradation), 6 samples out of 74 (8.1%) were excluded. As revealed by NGS analysis (Fig. 4d–f), a high percentage (47.1%) of anal adenocarcinoma harbored a *KRAS* mutation. In contrast, mutations in other genes were relatively rare (*NRAS* (5.9%), *BRAF* (1.5%), *PIK3CA* (7.6%)*, PTEN* (2.9%)) or completely absent (*EGFR*, *HER2*, *KIT*, *PDGFRA* and *DDR2*). No case had concurrent *KRAS* and *NRAS* mutations (Fig. 4d). Interestingly, when both tumour origin and HPV status were taken into account, the rate of *KRAS* mutations was significantly lower in anal gland/transitional-type neoplasms (8/24, 33.3%) (especially in HPV-positive tumours (2/10, 20%)) compared to their colorectal-type counterparts (24/44, 54.5%) (Fig. 4d, e). Finally, MSI was assessed and differences in the length of two or more mononucleotide repeats (indicative of MSI-high status) were only observed in one (1/74, 1.4%) anal adenocarcinoma (Fig. 4c and Supplementary Figure 3).

### The stroma surrounding anal gland/transitional-type anal adenocarcinoma is characterised by a prominent T-cell infiltration

**Fig. 5:**
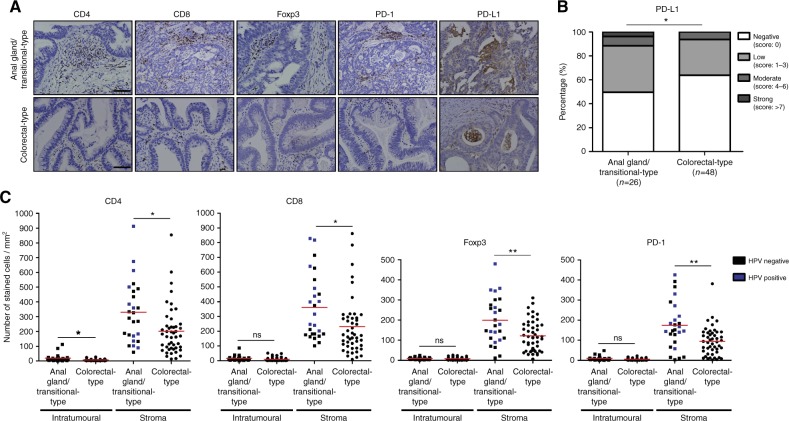
Topographic distribution and computerised quantification of infiltrating T-cell subsets in the tumour microenvironment. **a** Immunohistochemical analysis for CD4, CD8, Foxp3, PD-1 and PD-L1 was performed in both anal gland/transitional-type (*n* = 26) and colorectal-type (*n* = 48) anal adenocarcinoma. **b** Semiquantitative evaluation of PD-L1 expression in tumour cells. **c** CD4^+^, CD8^+^, Foxp3^+^, and PD-1^+^ cell infiltrations in both the epithelial component of the tumour and the stroma surrounding cancer cells were determined by computerised counting and verified manually. The number of positive cells was reported to tumour area yielding a count expressed as number of cells/mm^2^. The scale bar represents 100 μm. Asterisks indicate statistically significant differences (*p < 0.05; **p < 0.01)

Immunotherapy represents a great hope for many patients currently facing diseases associated with unfavorable outcomes and poor response rates to “classical” chemoradiotherapy. However, the therapeutic efficacy of drugs targeting the PD-1/PD-L1 axis (immune checkpoint inhibitors) relies on a high preexisting T-cell infiltration within tumour microenvironment. In order to determine whether anal glandular neoplasms meet this criteria, PD-L1 expression in epithelial (tumour) cells as well as intratumoural CD4^+^, CD8^+^, Foxp3^+^, and PD-1^+^ T cell densities were analysed by immunohistochemistry (Fig. 5a). These latter immune cell subsets were quantified by computerised counting (Supplementary Figure 4). Strikingly, higher T-cell infiltrates were observed in anal gland/transitional-type anal adenocarcinoma compared to tumours arising from the colorectal mucosa (Fig. 5c). Whatever the immune cell subpopulations, the large majority of positive cells (>90%) were detected in the stroma surrounding cancer cells. Within anal gland/transitional-type tumours, HPV-positive neoplasms were frequently associated with a highly inflammatory tumour microenvironment (Fig. 5c). Beside T-cell subsets, epithelial PD-L1 expression was also statistically more important in anal adenocarcinomas arising from the anal glands/transitional zone compared to their Krt20/CDX2-positive counterparts (Fig. 5b).

## Discussion

Anal adenocarcinoma remains a challenge for gastroenterologists, abdominal surgeons and oncologists because of its low incidence, unclear pathogenesis and high recurrence rate. So far, no epidemiologic, genetic and immunologic data are available and only a few studies (mainly case reports) focused on this rare and aggressive disease. Altogether, our findings indicate that the cellular origin (anal glands/transitional zone versus colorectal mucosa anatomically located under the anorectal flexure) of anal glandular neoplasms influences their natural history, mutational profile and immune microenvironment. Although the tumours were morphologically similar, keratin/CDX2 expression was first shown to be highly specific and sensitive for distinguishing the two adenocarcinoma subtypes described in the present article. The shared expression pattern detected in both normal tissues and invasive cancers further confirms the increasing use of keratin filaments as reliable biomarkers for correlating tumour origin with patient management.^30^ Moreover, the restricted expression of Krt7 in tumours arising from the anal glands/transitional zone is consistent with prior case reports^31–33^ and parallels the staining pattern of this biomarker in anal SCC located at the squamocolumnar junction.^2,3^

The carcinogenic potential of some HPV genotypes (most notably HPV16 and 18) was first reported in early 80s.^34^ Every year, about 630,000 SCC attributable to HPV infection are diagnosed worldwide.^35^ These latter are mainly diagnosed in the uterine cervix but increasing incidences are currently observed in both anal canal and oropharynx. Frequently under-estimated by both virologists and epidemiologists, carcinogenic HPV genotypes are also involved in the development of the majority (~75–80%) of cervical adenocarcinoma.^36^ Interestingly, transcriptionally active HPV infections were recently found in Barrett’s adenocarcinoma,^37^ another glandular neoplasm developing within a squamocolumnar junction. To the best of our knowledge, this report provides the first evidence of a causal link between HPV infection and anal adenocarcinoma. Similarly to other HPV-positive adenocarcinoma diagnosed in the uterine cervix or oesophagus, only anal glandular neoplasms (11/26, 42.3%) arising from the transitional zone were infected by HPV16 or 18. Although several possible mechanisms were recently highlighted,^38^ the high susceptibility of junctional/transitional tissues to HPV infection is still the subject of active investigations. Despite a few false-positive (p16^ink4^ positive/HPV negative) results (4/63, 6.3%) that were also shown in the context of both anal and oropharyngeal SCC,^2,6,39^ the reported correlation between high-risk HPV infection and diffuse p16^ink4^ immunoreactivity was expected. The surprising result was that five (5/11, 45.5%) HPV-infected tumours displayed some patches (representing up to one third of the whole tumour area) of p16^ink4^ negativity. Given that aberrant methylation of *p16*^ink4^ promoter is frequently observed in adenocarcinoma arising from the gastrointestinal tract,^40,41^ MSP experiments were performed and *CDKN2A* (*p16*^ink4^) promoter hypermethylation was detected in 4 out of 5 tumour samples (Supplementary Figure 5).

During the last 20 years, local excision, combined chemoradiotherapy and radical surgery with or without (neo)adjuvant chemotherapy have been successively proposed as the gold standard treatment for these aggressive anal glandular tumours.^16^ Unfortunately, conflicting results exist due to the low number of followed patients in most studies.^16^ To date, only one study conducted by the Rare Cancer Network analysed more than 50 patients and observed better survival rates after combined chemoradiotherapy compared to radiotherapy/surgery. However, patients treated with this latter combination were significantly older, making difficult the comparison between the two groups.^9^ Accordingly, our findings demonstrated that patient age was an independent predictor for decreased OS. Therefore, with the exception of local surgery for stage I tumour, as reported in the present study and suggested by the discrepancies in the literature, no standard treatment was shown to clearly improve patient outcome.

First suggested by Paul Ehrlich one century ago, the concept of targeted therapy has gained momentum since early 2000s with the clinical success of inhibitors such as trastuzumab (for both breast and gastric cancers) or imatinib (for chronic myeloid leukaemia).^42^ Treatment algorithms of numerous cancers have changed dramatically over the last few years due to the advent of novel targeted drugs. In the context of cancers diagnosed within the gastrointestinal tract, two anti-EGFR monoclonal antibodies (cetuximab and panitumumab) in combination with FOLFIRI were recently approved for the first-line treatment of metastatic colorectal cancers.^43,44^ However, their efficacy strongly depends on both EGFR expression and mutational status of its downstream effectors (*KRAS*, *NRAS*, *BRAF*, *PI3KCA*, *PTEN*).^28,29^ While EGFR expression was detected in the large majority (64/74, 86.5%) of both subtypes of anal adenocarcinoma irrespective of the HPV status, interestingly, the frequencies of *KRAS*/*NRAS* mutations were significantly higher in HPV-negative tumours compared to their infected counterparts. The low rates of both *KRAS* and *NRAS* mutations reported in neoplastic lesions etiologically linked to HPV concurred with the recent results documented for cervical adenocarcinoma.^45^ Regarding the low prevalence of both *BRAF* mutations and MSI-high specimens observed in the present study, these latter results are in agreement with those of Yamauchi et al.^46^ who showed a gradual decrease of these two parameters along the gastrointestinal tract from the ascending colon to the rectum.

Chosen by Science’s editors as the breakthrough of the year for 2013,^47^ cancer immunotherapy using immune checkpoint inhibitors (especially drugs targeting the PD-1/PD-L1 axis) has raised much interest for cancer treatment over the last few years. However, their efficacy for reactivating lymphocyte-mediated cytotoxicity relies on a high pre-existing T cell infiltration of the tumour microenvironment. Importantly, we reported here that T cell densities (CD4, CD8, Foxp3 and PD-1) detected in the tumour stroma surrounding cancer cells (especially HPV-positive ones) originating from the anal glands/transitional zone were significantly higher than those found in the vast majority of colorectal-type tumours. Moreover, a similar significant increase was also observed for the expression of PD-L1 by tumour cells. Not related to the MSI status of tumours, these data are likely to be explained by the chronic inflammation frequently observed in squamocolumnar junctions^48^ and/or by the detection of transcriptionally active HPV infection in approximately half (42.3%) of anal gland/transitional-type adenocarcinoma. Indeed, several recent studies described a significant increased density of both effector (CD4/CD8) and regulatory (Foxp3) tumour infiltrating T lymphocytes in HPV-positive compared to HPV-negative SCC and highlighted the predictive value of CD4/CD8 infiltrates.^49^ These latter results and those reported in the present article are supportive of the dozen ongoing clinical trials evaluating immune-checkpoint inhibitors in the setting of HPV-positive cancers.

Taken together, our findings highlight the existence of two subtypes of anal adenocarcinoma with distinct cells of origin. Whatever the tumour subtypes, metastatic patients with wild-type downstream effectors of the EGFR signalling pathway could reasonably be expected to benefit from anti-EGFR monoclonal antibodies. Displaying a strong Krt7 immunoreactivity, tumours arising from the anal glands/transitional zone were shown to be associated with prominent T cell infiltrates and high expressions of PD-1/PD-L1 suggesting that immune checkpoint inhibitors could also represent promising alternatives to current sub-optimal treatment algorithms. Although further studies are still needed, determining the cellular origin of these rare tumours with the specific/sensitive biomarkers validated in the present study could therefore be helpful for clinicians currently struggling with this aggressive neoplastic condition.

## Electronic supplementary material

Supplementary material and methods

Supplementary table 1

Supplemental Figure 1

Supplemental Figure 2

Supplemental Figure 3

Supplemental Figure 4

Supplemental Figure 5
