# Supplementary material for: A dualistic model of primary anal canal adenocarcinoma with distinct cellular origins, etiologies, inflammatory microenvironments and mutational signatures: implications for personalised medicine
Source: Br J Cancer. 2018 Apr 27;118(10):1302–12. doi: 10.1038/s41416-018-0049-2 (PMC5959925; doi:10.1038/s41416-018-0049-2)
Supplement: Supplementary file 1 — Supplementary material and methods [file 41416_2018_49_MOESM1_ESM.docx]

**Supplementary Material and Methods**

*HPV physical status determination*

HPV16 and 18 physical status (episomal, mixed, integrated) was determined through HPV E2/E6 ratio analysis. For quantitative real-time PCR experiments [ABI-Prism 7900 Sequence Detection System (Applied Biosystems, Foster City, CA, USA)], 20 ng of cDNA were amplified in 25 μl of 1× FastStart SYBR Green Master mix (Roche, Bale, Switzerland) containing 200 or 300 nmol/L of each primer. Thermal cycling conditions were: 50°C for 2 minutes, 95°C for 10 minutes, 40 cycles of denaturation at 95°C for 30 seconds, annealing at 60°C for 1 minute and extention at 72°C for 1 minute. Each real-time PCR experiment was performed in triplicate and normalized to the amount of GAPDH mRNA from the same sample. The cut-off value was determined as previously described. Primer sequences were as follows:

HPV16 E2 forward: AAG GCG ACG GCT TTG GTA T

HPV16 E2 reverse: GCA ACG AAG TAT CCT CTC CT

HPV16 E6 forward: CTG CGA CGT GAG GTG TAT TAA C

HPV16 E6 reverse: TGG AAT CTT TGC TTT TTG TCC

HPV18 E2 forward: TGC AAG ACA CAT GCG AGG AA

HPV18 E2 reverse: CAT GTT CCT GCA TCA GTC ATA T

HPV18 E6 forward: CTA TAG AGG CCA GTG CCA TTC G

HPV18 E6 reverse: TTA TAC TTG TGT TTC TCT GCG TCG

GAPDH forward: ACC AGG TGG TCT CCT CTG AC

GAPDH reverse: TGC TGT AGC CAA ATT GGT TG

*Microsatellite instability (MSI) assessment*

Each collected tumor specimen was carefully reviewed by a histopathologist in order to ascertain that the percentage of neoplastic cell was higher than 40%. When tumor cell enrichment was necessary, tumor areas were macrodissected with a scalpel before tissue digestion. The number of sections per case varied from 3 to 10 according to sample size. Genomic DNA was extracted using QIAamp DNA FFPE Tissue Kit (Qiagen, Valencia, CA, USA) according to the manufacturer's recommendations. Thermal cycling conditions were: 95°C for 5 minutes, 35 cycles of denaturation at 95°C for 30 seconds, annealing at 55°C for 30 seconds and extension at 72°C for 30 seconds, followed by a final extension at 72°C for 5 minutes. PCR products labeled with fluorescent dyes (FAM for BAT-26 and NR-21, HEX for BAT-25 and NR-27 and NED for NR-24) were analyzed with an ABI 3500xL Genetic Analyzer (Applied Biosystems). As recommended by the revised Bethesda guidelines, tumors displaying differences in the length of two or more microsatellite sequences (markers) were interpreted as being MSI-High. Lesions showing no instability or one instable repeat were defined as microsatellite stable (MSS) and MSI-Low, respectively. As previously described by Buhard et al., primer sequences were as follows:

*hMSH2*

BAT-26 forward: CTG CGG TAA TCA AGT TTT TAG

BAT-26 reverse: AAC CAT TCA ACA TTT TTA ACC C

*c-kit*

BAT-25 forward: TAC CAG GTG GCA AAG GGC A

BAT-25 reverse: TCT GCA TTT TAA CTA TGG CTC

*Zinc finger 2*

NR-24 forward: GCT GAA TTT TAC CTC CTG AC

NR-24 reverse: ATT GTG CCA TTG CAT TCC AA

*SLC7A8*

NR-21 forward: GAG TCG CTG GCA CAG TTC TA

NR-21 reverse: CTG GTC ACT CGC GTT TAC AA

*Inhibitor of apoptosis Protein-1*

NR-27 forward: AAC CAT GCT TGC AAA CCA CT

NR-27 reverse: CGA TAA TAC TAG CAA TGA CC

*Mutation analysis using next-generation sequencing (NGS)*

Regions of interest from 10 clinically relevant genes in the gastointestinal cancer setting {*KRAS* [exons 2, 3 and 4 (full)], *NRAS* [exons 2, 3 and 4 (full)], *BRAF* [exons 11 and 15 (full)], *PIK3CA* [hotspots in exon 9 and 20 (codons 542, 545 and 1047 covered)], *EGFR* [exons 18, 19, 20 and 21 (full)], *HER2* [exon 22 (full)], *KIT* [exons 9, 11, 13, 14, 17 and 18 (full)], *PTEN* [exons 5 and 7 (full)], *PDGFRA* [exons 12, 14 and 18 (full)], *DDR2* [exon 18 (full)]} were amplified by multiplex PCR using Qiagen multiplex PCR plus (Qiagen, Valencia, CA, USA). All PCR conditions and primer sequences are available upon request. Molecular barcoding was performed with the MID kit for Illumina Miseq (Multiplicom, Niel, Belgium) according to the manufacturer’s recommendations. PCR products of each patient were purified using Agencourt AMPure XP beads (Beckman Coulter, Brea, CA, USA) and then quantified using Quant-iT PicoGreen dsDNA Assay Kit (Fisher Scientific, USA) and the BMG FLUOstar OPTIMA Microplate Reader (BMG Labtech, Ortenberg, Germany). These individually tagged amplicon libraries were pooled in equimolar amounts in order to obtain the final library. This latter was then sequenced on the Illumina MiSeq sequencing platform using a MiSeq v2 cartridge (500 cycles). Data were finally analyzed using the SeqNext module (version 4.1.1) (JSI Medical systems, Kippenheim, Germany).
