# Supplementary material for: A dualistic model of primary anal canal adenocarcinoma with distinct cellular origins, etiologies, inflammatory microenvironments and mutational signatures: implications for personalised medicine
Source: Br J Cancer. 2018 Apr 27;118(10):1302–12. doi: 10.1038/s41416-018-0049-2 (PMC5959925; doi:10.1038/s41416-018-0049-2)
Supplement: Supplementary file 2 — Supplementary table 1 [file 41416_2018_49_MOESM2_ESM.docx]

| **Supplementary table 1. Demographic and patient characteristics for anal gland/transitional-type adenocarcinoma according to HPV status** | | | | |
| --- | --- | --- | --- | --- |
|  |  |  |  |  |
| **Characteristics** |  | **HPV-positive anal gland/transitional-type (n=11) (42.3%)** | **HPV-negative anal gland/transitional-type (n=15) (57.7%)** | ***P*-Value** |
| **Age at diagnosis** (mean: 63)(range: 36-94) (years) | |  |  |  |
| < 65 |  | 7 (63.6%) | 5 (33.3%) | 0.233 |
| ≥ 65 |  | 4 (36.4%) | 10 (66.7%) |  |
| **Gender** |  |  |  | 1 |
| male |  | 6 (54.5%) | 8 (53.3%) |  |
| female |  | 5 (45.5%) | 7 (46.7%) |  |
| **Inflammatory bowel disease** |  |  |  | 0.614 |
| negative |  | 10 (90.9%) | 12 (80%) |  |
| positive |  | 1 (9.1%) | 3 (20%) |  |
| **HIV infection** |  |  |  |  |
| negative |  | 11 (100%) | 15 (100%) | / |
| positive |  | 0 (0%) | 0 (0%) |  |
| **Proliferative index (Ki67)** |  |  |  | 0.389 |
| ≤ 25% |  | 3 (27.3%) | 4 (26.7%) |  |
| 26-50% |  | 3 (27.3%) | 8 (53.3%) |  |
| 51-75% |  | 4 (36.3%) | 3 (20%) |  |
| >75% |  | 1 (9.1%) | 0 (0%) |  |
| **Tumor differentiation** |  |  |  | 0.908 |
| well-differentiated |  | 4 (36.3%) | 5 (33.3%) |  |
| moderately differentiated |  | 5 (45.5%) | 8 (53.3%) |  |
| poorly differentiated |  | 2 (18.2%) | 2 (13.4%) |  |
| **cTNM** |  |  |  |  |
| cT |  |  |  | 0.453 |
| T1-T2 |  | 7 (63.7%) | 7 (46.7%) |  |
| T3-T4 |  | 4 (36.3%) | 8 (53.3%) |  |
| cN |  |  |  |  |
| N- |  | 5 (45.5%) | 8 (53.3%) | 1 |
| N+ |  | 6 (54.5%) | 7 (46.7%) |  |
| cM |  |  |  | 0.169 |
| M- |  | 9 (81.8%) | 15 (100%) |  |
| M+ |  | 2 (18.2%) | 0 (0%) |  |
| **Tumor stage (UICC)** |  |  |  | 0.377 |
| stage I |  | 1 (9.1%) | 1 (6.6%) |  |
| stage II |  | 4 (36.3%) | 7 (46.7%) |  |
| stage III |  | 4 (36.3%) | 7 (46.7%) |  |
| stage IV |  | 2 (18.2%) | 0 (0%) |  |
| **Primary treatment** |  |  |  | 0.422 |
| (neo)adjuvant chemoradiotherapy/surgery |  | 4 (36.3%) | 7 (46.7%) |  |
| (neo)adjuvant radiotherapy/surgery |  | 2 (18.2%) | 2 (13.3%) |  |
| (neo)adjuvant chemotherapy/surgery |  | 1 (9.1%) | 0 (0%) |  |
| chemoradiotherapy |  | 0 (0%) | 2 (13.3%) |  |
| radiotherapy |  | 1 (9.1%) | 2 (13.3%) |  |
| chemotherapy |  | 2 (18.2%) | 0 (0%) |  |
| surgery |  | 1 (9.1%) | 2 (13.3%) |  |
| no treatment |  | 0 (0%) | 0 (0%) |  |
