# Supplementary figures and images for: A dualistic model of primary anal canal adenocarcinoma with distinct cellular origins, etiologies, inflammatory microenvironments and mutational signatures: implications for personalised medicine
Source: Br J Cancer. 2018 Apr 27;118(10):1302–12. doi: 10.1038/s41416-018-0049-2 (PMC5959925; doi:10.1038/s41416-018-0049-2)

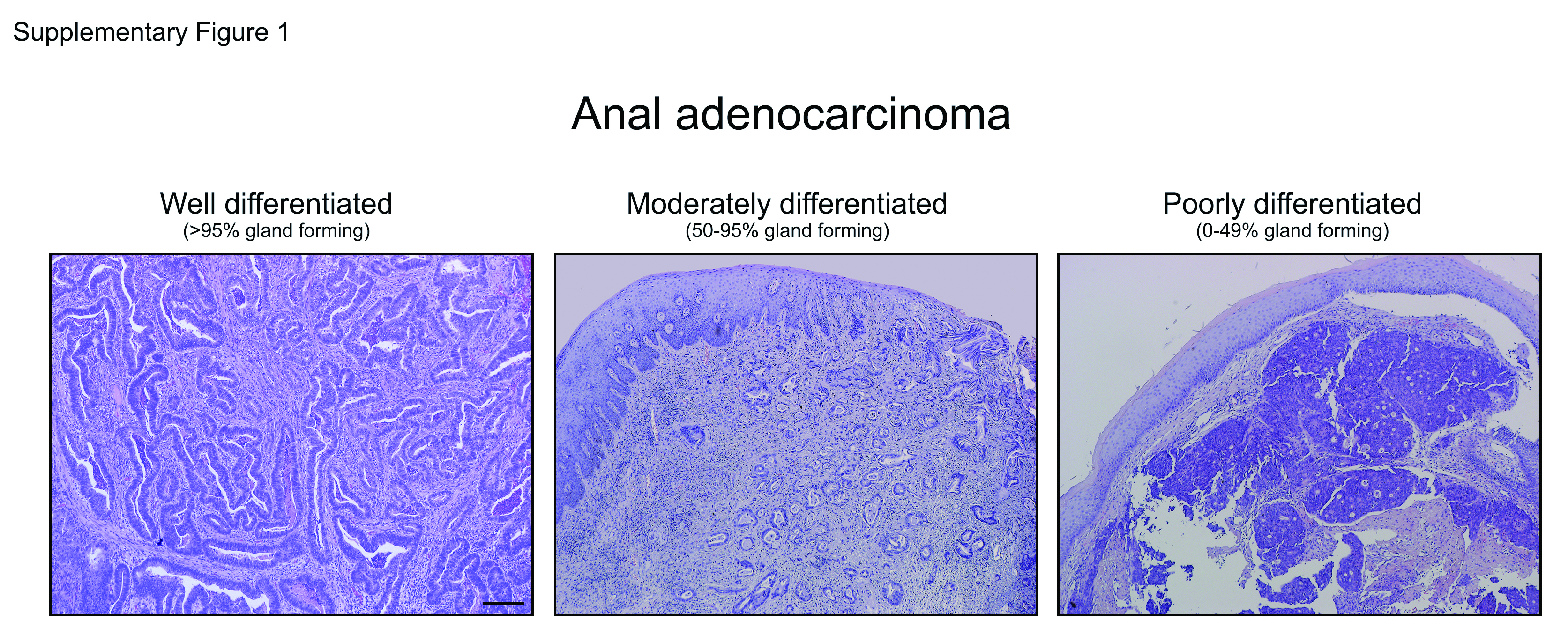

Supplement: Supplementary file 3 — Supplemental Figure 1 [file 41416_2018_49_MOESM3_ESM.jpg]

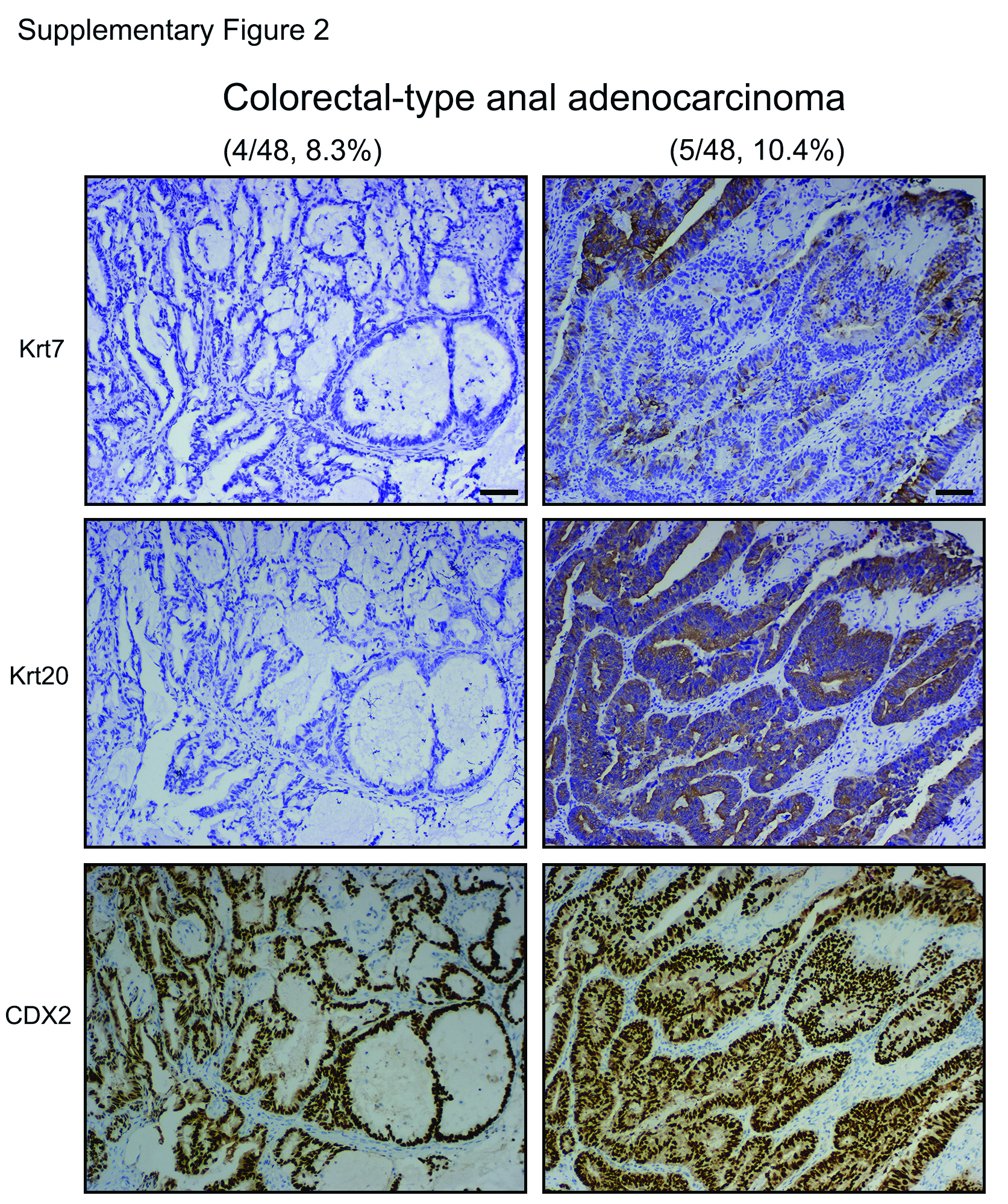

Supplement: Supplementary file 4 — Supplemental Figure 2 [file 41416_2018_49_MOESM4_ESM.jpg]

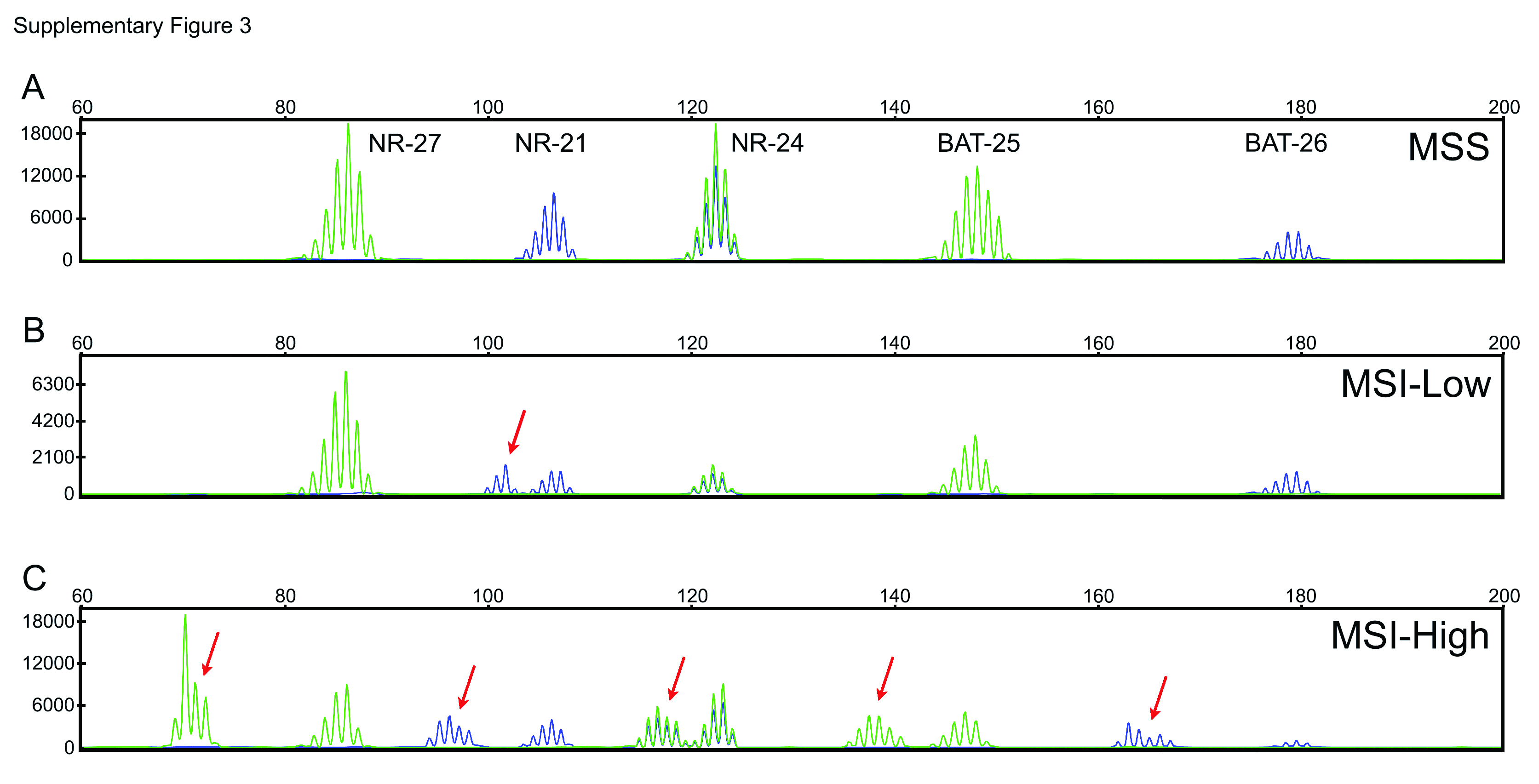

Supplement: Supplementary file 5 — Supplemental Figure 3 [file 41416_2018_49_MOESM5_ESM.jpg]

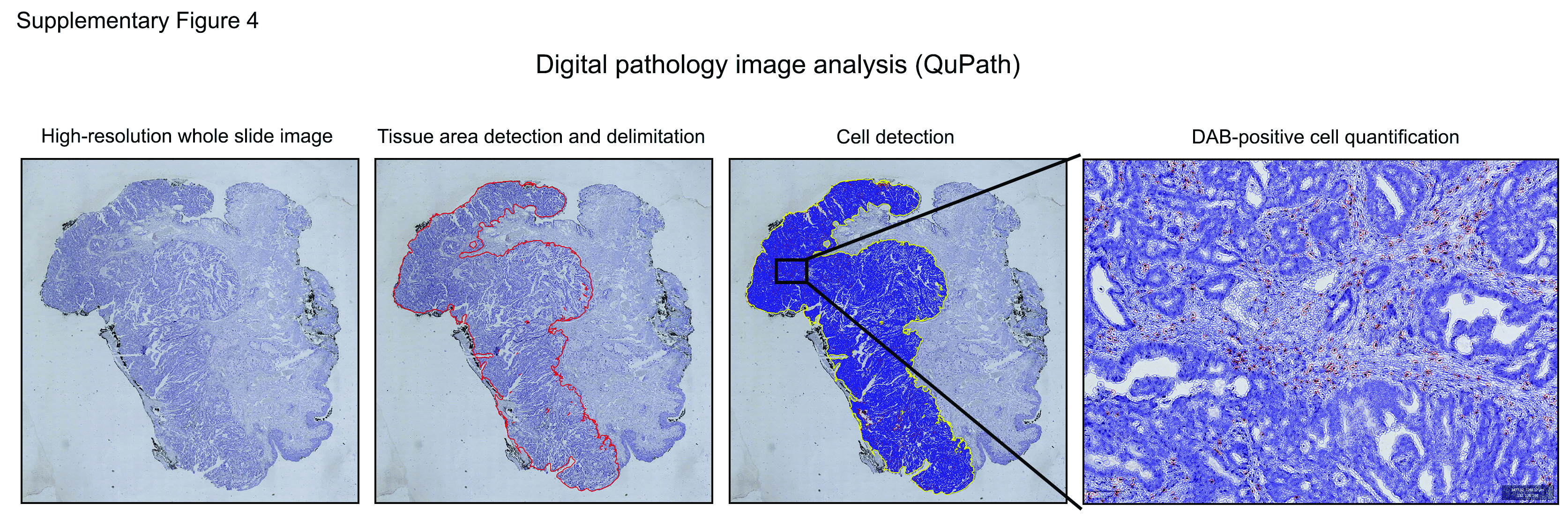

Supplement: Supplementary file 6 — Supplemental Figure 4 [file 41416_2018_49_MOESM6_ESM.jpg]

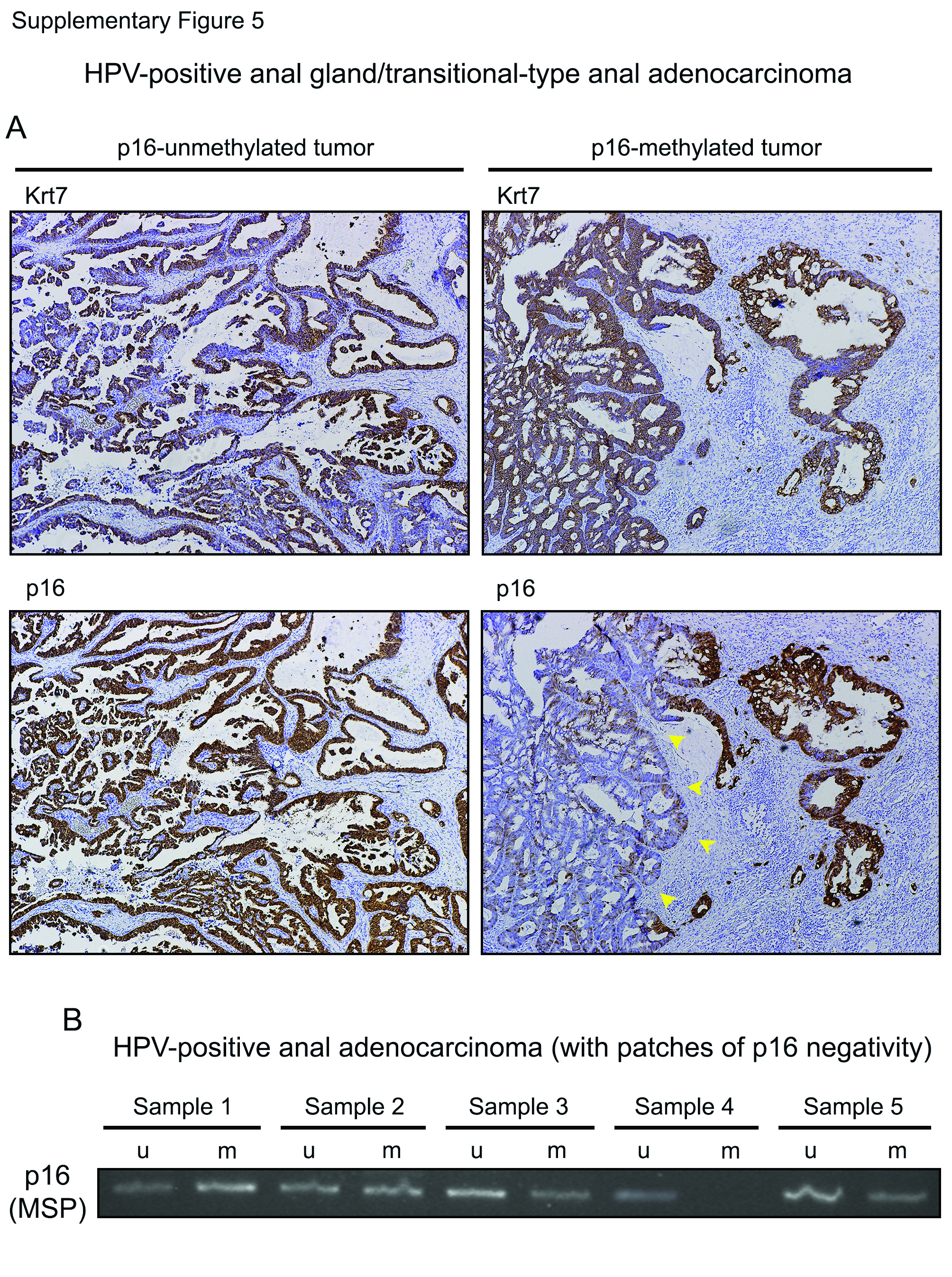

Supplement: Supplementary file 7 — Supplemental Figure 5 [file 41416_2018_49_MOESM7_ESM.jpg]
